# Identification of *AUXIN RESPONSE FACTOR* gene family from *Prunus sibirica* and its expression analysis during mesocarp and kernel development

**DOI:** 10.1186/s12870-017-1220-2

**Published:** 2018-01-24

**Authors:** Jun Niu, Quanxin Bi, Shuya Deng, Huiping Chen, Haiyan Yu, Libing Wang, Shanzhi Lin

**Affiliations:** 1 Hainan Key Laboratory of Sustainable Utilization of Tropical Bioresources, Institute of Tropical Agriculture and Forestry, Hainan University, Haikou, Hainan 570228 China; 2 State Key Laboratory of Tree Genetics and Breeding, Research Institute of Forestry, Chinese Academy of Forestry, Beijing, 100091 China; 3 Key Laboratory of Genetics and Breeding in Forest Trees and Ornamental Plants, College of Biological Sciences and Biotechnology, Beijing Forestry University, Beijing, 10083 China

**Keywords:** *Prunus Sibirica*, Auxin response factor, Expression profile, Co-expression analysis

## Abstract

**Background:**

Auxin response factors (ARFs) in auxin signaling pathway are an important component that can regulate the transcription of auxin-responsive genes involved in almost all aspects of plant growth and development. To our knowledge, the comprehensive and systematic characterization of *ARF* genes has never been reported in *Prunus sibirica*, a novel woody biodiesel feedstock in China.

**Results:**

In this study, we identified 14 *PsARF* genes with a perfect open reading frame (ORF) in *P. sibirica* by using its previous transcriptomic data. Conserved motif analysis showed that all identified PsARF proteins had typical DNA-binding and ARF domain, but 5 members (PsARF3, 8 10, 16 and 17) lacked the dimerization domain. Phylogenetic analysis of the ARF proteins generated from various plant species indicated that ARFs could be categorized into 4 major groups (Class I, II, III and IV), in which all identified ARFs from *P. sibirica* showed a closest relationship with those from *P. mume*. Comparison of the expression profiles of 14 *PsARF* genes in different developmental stages of Siberian apricot mesocarp (SAM) and kernel (SAK) reflected distinct temporal or spatial expression patterns for *PsARF* genes. Additionally, based on the expressed data from fruit and seed development of multiple plant species, we identified 1514 *ARF*-correlated genes using weighted gene co-expression network analysis (WGCNA). And the major portion of *ARF*-correlated gene was characterized to be involved in protein, nucleic acid and carbohydrate metabolic, transport and regulatory processes.

**Conclusions:**

In summary, we systematically and comprehensively analyzed the structure, expression pattern and co-expression network of *ARF* gene family in *P. sibirica*. All our findings provide theoretical foundation for the *PsARF* gene family and will pave the way for elucidating the precise role of *PsARF* genes in SAM and SAK development.

**Electronic supplementary material:**

The online version of this article (10.1186/s12870-017-1220-2) contains supplementary material, which is available to authorized users.

## Background

Siberian apricot (*Prunus sibirica* L.), belonging to family Rosaceae, is diploid plant: 2n = 2× = 16 [29]. Recently, Siberian apricot has become a novel and important woody oilseed plant. In China, the total area of Siberian apricot is approximately 1.7 million ha, and the annual harvest of seeds is nearly 192,500 tons [48]. Siberian apricot is a multipurpose tree species with ecological and economic value. The Siberian apricot mesocarp (SAM) can be eaten either dried or fresh. The Siberian apricot kernel (SAK) was identified with high content of oil (over 50%), and the SAK oil was determined to be suitable for biodiesel production based on evaluation of cold filter plugging point, cetane number, oxidative stability and flash point [49]. Recently, the investigations of morphological characteristics and oil contents in developing SAK revealed that the development of SAK is a dynamic process involving a complex series of specific respond to different developmental signals [11, 32].

Increasing evidences suggested the ubiquitous involvement of auxin in transcriptional regulation of various genes, which may function in most aspects of growth and development processes, such as embryogenesis, organogenesis, tissue differentiation, apical dominance, root initiation, flower, fruit and seed development [6, 7, 25]. Numerous studies in *Arabidopsis* and other plant species elucidated that these auxin-inducible genes contain auxin response element (AuxRE: TGTCTC) in their promoters, and auxin response factors (ARFs) involved in auxin perception and signaling could specifically bind AuxRE [18, 20, 24, 25, 42, 47, 55]. The typical ARF proteins contain 3 representative components, an N-terminal DNA-binding domain (DBD), a middle domain (MD) and a carboxy-terminal dimerization domain (CTD) [6]. The DBD domain, as a plant-specific B3-type protein domain, is responsible for the recognition of AuxRE in the promoter of auxin-responsive genes. The CTD domain, consisting of two highly conserved dimerization domains III and IV, similar to those found in auxin/indole-3-acetic acid (Aux/IAA) proteins [17, 18]. The MD, in the middle region between the DBD and CTD, functions as an activation domain (AD) or repression domain (RD) to regulate the expression of downstream genes [12]. AD is enriched in glutamine (Q) residues, while RD is enriched in proline (P), serine (S) and threonine (T) residues [6].

Since, cloning of the first *ARF1* gene from *Arabidopsis* [46], genome-wide analyses have identified *ARF* gene family from 29 plant species [6]. For example, 23 genes from *Arabidopsis* [18], 35 genes from *Gossypium raimondi* [42], 22 genes from *Citrus sinensis* [24], 17 genes from *Eucalyptus grandis* [55], 15 genes from *Cucumis sativus* [27], 36 genes from *Zea mays* [28], 25 genes from *Oryza sativa* [47] and 39 genes from *Populus trichocarpa* [20] have been defined*.* The quantity of *ARF* genes in different plant species were obviously different, which could be due to extensive duplication and diversification in the evolution of these plants [3]. Interestingly, most ARF proteins from these plant species are nuclear proteins, and maintain consistency of conserved domains as above described [6, 25]. Although wide analysis of ARFs function, expression, and regulation has been performed in annual herbaceous plants, relatively few reports focus on perennial woody plants [25].

To date, biochemical and genetic approaches have enabled identifying *ARF* gene functions in development of plant seeds and fruits. For example, mutation analyses revealed that *AtARF2* is a general repressor of cell division to regulate seed size and weight [38]. The mutation of *AtARF5* in *Arabidopsis* suggested that the *AtARF5* gene influences embryo pattern formation as well as vascular development by mediating axialized behavior of plant cells in response to auxin cues [19]. T-DNA insertion alleles demonstrated that *AtARF8* is an important regulator of fruit initiation and that the disruption of its normal function induces parthenocarpy in *Arabidopsis* [14]. In tomato (*Solanum lycopersicon*), *SlARF4* was demonstrated to be involved in sugar metabolism and cell wall architecture during tomato fruit development [36, 37]. The transgenic antisense-*OsARF1* rice showed extremely low growth, poor vigor, short curled leaves and sterility, suggesting that the *OsARF1* is essential for growth in vegetative organs and seed development [1]. Together, these studies have shown that the *ARF* gene family is widely involved in regulating growth and development of seeds and fruits.

Here, we identified *ARF* genes from the previous transcriptomic data of Siberian apricot, and analyzed their phylogenetic relationship, gene structure and protein motifs. To understand the contribution of *PsARF*s in SAM and SAK development, we also characterized the expression profiles and co-expression networks of *PsARF* genes. Our present work is a necessary step in formulating further studies of the function of *PsARF* genes and the ARF-mediated auxin signaling pathway in growth and development of Siberian apricot.

## Methods

### Plant material

The different developmental stages of Siberian apricot were obtained from the same tree located at the Beijing Forestry University experimental station, Beijing, China. The developmental processes of Siberian apricot from flowering to seed maturity were observed from May to July 2017. Flowers with the same anthesis were marked, and then fruits were respectively harvested at 10, 30, 50, 60, and 70 days after flowering (DAF), based on our previous report [31]. The SAM and SAK in different development periods were immediately separated and frozen in liquid nitrogen, and stored at −80 °C until use.

### *PsARF* sequence retrieval and conserved motif analysis

Our data were obtained from the previous transcriptomic studies of SAM and SAK at 10, 30, 50, 60, and 70 DAF by Illumina sequencing (PRJNA260249) [31], and a mixture of buds, leaves, stems, flowers, fruits by 454 pyrosequencing (SRX339392) [9]. After removal of the adapter sequences, the low-quality sequences (reads with ambiguous bases ‘N’) and reads with more than 10% Q < 20 bases, the clean reads were assembled into unigenes with the Trinity program [53]. A total of 124,070 unigenes (N50: 1603 bp) with the mean length of 829.62 bp was obtained. The Siberian apricot unigenes were annotated using BLASTX alignment with an E-value cut-off of 10^−5^ against the following protein databases: *Arabidopsis* proteome (www.arabidopsis.org), NCBI nonredundant (https://www.ncbi.nlm.nih.gov/) and SwissProt (www.uniprot.org). Based on the annotated results, the unigenes with ARF family domain were filter out for the prediction of open reading frame (ORF) by NCBI ORF finder (https://www.ncbi.nlm.nih.gov/orffinder/). All non-overlapping *PsARF* genes with a perfect ORF were validated by PCR amplification and gene sequencing, and also were used for further analysis. The primer sequences of PCR amplification are shown in Additional file 1: Table S1.

To exhibit the structural divergence of PsARF proteins, the conserved motifs were performed with Multiple Expectation Maximization for Motif Elicitation (MEME) 4.11.2 online program [2]. The following parameters were employed in the analysis: the maximum number of motifs 20; minimum motif width 6; and maximum motif width 50. A phylogenetic tree of PsARF proteins was constructed with the MEGA 7.0 software.

### Phylogenetic analysis *PsARF* genes

For phylogenetic reconstruction of the *ARF* gene family, we downloaded *ARF* genes from the sequenced genomes, including *Arabidopsis* (23) [18], *Brassica rapa* (31) [30], *Citrus sinensis* (19) [24] and *P. mume* (17) [41]. Amino acid sequences of the *PsARF* genes were aligned using ClustalW with default options [43], and the alignment was manually corrected at both ends to eliminate regions of poor alignment by using Jalview [51]. Phylogenetic trees were constructed by the neighbor-joining method using program MEGA 7.0 [21]. The parameters of the constructed trees were: phylogeny test and options, bootstrap (1000 replicates); gaps/missing data, complete deletion; model, amino acid; Poisson correction; substitutions to include, all; pattern among lineages, same (homogeneous); and rates among sites, uniform rates.

### Co-expression analysis of *PsARF*

To precisely determine genes whose expression tightly correlates with that of *ARF* genes in fruit and seed development, a series of expression data was analyzed derived from *Elaeis guineensis* [4], *P. sibirica* [31], *B. napus*, *Ricinus communis*, and *Euonymus alatus* [44]. The expression data contains 5, 5, 4, 4, 4, 5 and 5 developmental phases of oil palm mesocarp, date palm mesocarp, *B. napus* seed, *R. communis* seed, *E. alatus* seed, SAM and SAK, respectively. Automatic construction of the gene network and identification of modules were conducted by using the R package of weighted gene co-expression network analysis (WGCNA) [22]. By pickSoftThreshold analysis, the power 12 was selected to amplify the strong connections between genes and penalize the weaker connections. Here, we used a convenient one-step network construction and module detection, and chose a relatively large minimum module size of 30 and a medium sensitivity (DeepSplit = 2) to cluster splitting [22]. The resulting genes (threshold weight ≥ 0.1) involved in PsARFs was analyzed with BGI WEGO (http://wego.genomics.org.cn/) to assign gene ontology (GO) terms [54]. Cytoscape was implemented for visualizing interaction networks [39].

### Expressed analysis by FPKM and qRT-PCR

The recent development of the RNA-Seq method provides information on wide and sensitive gene expression. In our RNA-seq data, the expression levels were calculated by Fragment Per Kilobase of exon model per Million mapped reads (FPKM). From these results, we extracted the expression data related to *PsARF* gene family, and conducted further analysis.

The equal weight of three biological samples of SAM and SAK in different developmental stages was mixed. And then total RNA of SAM and SAK was separately extracted from the mixture using RNeasy Plant Mini Kits (Qiagen) according to the manufacturer’s protocol. Three biological repetitions were performed for each RNA extraction. The fist-strand cDNA was synthesized by using oligo d(T) primers and reverse transcription System (Promega). The amplification primers were designed using PrimerQuest (http://www.idtdna.com/PrimerQuest/Home/Index) software with melting temperatures at 62 °C, and the absence of secondary structures was verified by the UNAFold program (http://eu.idtdna.com/UNAFold). According to our previous studies, cyclophilin and ubiquitin-conjugating enzyme were used as internal controls [33]. The qRT-PCR was performed using the SYBR Premix Ex Taq Kit (TaKaRa) according to the manufacturer’s protocol. Negative controls consisting of nuclease-free water instead of template, and reverse transcriptase controls prepared by substituting reverse transcriptase for nuclease-free water in the cDNA synthesis step were included in all analyses for each primer pair. Three technical repetitions were performed for qRT-PCR. The qRT-PCR primer sequences are shown in Additional file 1: Table S1. The statistical analysis of expressed data was performed using SPSS software.

## Results

### Identification of *PsARF* gene families in *P. sibirica*

To comprehensively identify the *PsARF* genes in *P. sibirica*, we integrated the transcriptomic data of *P. sibirica* form different developing kernels by Illumina/Solexa sequencing [31] and different tissues by 454 GS FLX Titanium sequencing [9]. Simultaneously, the 23 protein sequences of ARFs from the TAIR 10 (The *Arabidopsis* Information Resource) database were used as queries to search PsARFs in this work. After PCR amplification and gene sequencing (Additional file 2: Figure S1), we characterized 14 *PsARF* genes with a perfect ORF (Table 1). The minimum ORF length of *PsARF*s is 1803 bp (PsARF17) encoding 600 amino acid residues (66.23 kDa), and the maximum ORF length of *PsARF*s is 3489 bp (PsARF19) encoding 1162 amino acid residues (130.01 kDa) (Table 1). It is worth noting that the theoretical PI values of all PsARF proteins were less than 7 (Table 1), suggesting that those *PsARF* genes encoded weakly acidic proteins. The nomenclature system for *PsARF*s in the present study was given to the homologies of *Arabidopsis* ARFs.

**Table 1:** *ARF* genes in *P. sibirica*

Genes	NCBI ID	ORF length (bp)	Deduced polypeptide			TIGR locus ID	E-value
			Length (aa)	MW (kDa)	PI		
PsARF1	MF373590	2037	678	75.47	6.00	AT1G59750	0
PsARF2	MF373591	2540	846	94.26	6.55	AT5G62000	0
PsARF3	MF373592	2160	719	78.50	6.19	AT2G33860	e-174
PsARF4	MF373593	2406	801	88.47	5.96	AT5G60450	0
PsARF5	MF373594	2865	954	104.29	5.27	AT1G19850	0
PsARF6	MF373595	2670	889	98.46	6.11	AT1G30330	0
PsARF7	MF373596	3423	1140	126.22	6.19	AT5G20730	0
PsARF8	MF373597	2172	723	80.56	6.16	AT5G37020	0
PsARF9	MF373598	2046	681	75.34	6.20	AT4G23980	0
PsARF10	MF373599	2025	674	74.69	6.46	AT2G28350	0
PsARF11	MF373600	2097	698	77.41	6.37	AT2G46530	0
PsARF16	MF373601	2061	686	75.94	6.66	AT4G30080	0
PsARF17	MF373602	1803	600	66.23	6.52	AT1G77850	e-145
PsARF19	MF373603	3489	1162	130.01	6.20	AT1G19220	0

### Conserved motifs and domains in PsARF proteins

**Fig. 1:**
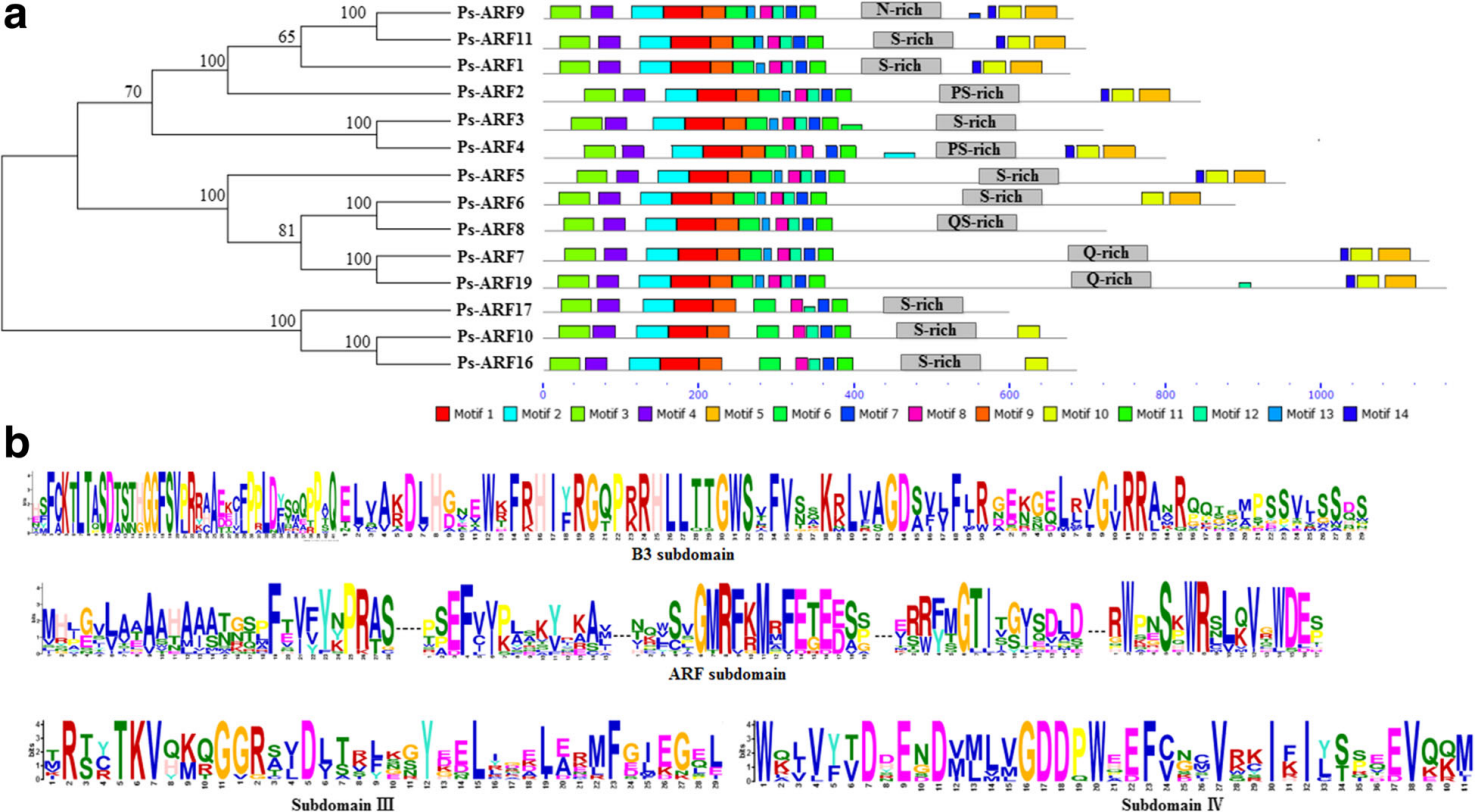
Diagrams and predicted sequences of PsARFs in *P. sibirica*. **a** Schematic diagrams of 14 PsARFs. **b** Amino acid sequence alignments of B3-type subdomain, ARF subdomain, subdomain III and subdomain IV

By using MEME online program, a total of 14 motifs was characterized from 14 PsARF proteins (Fig. 1a and Additional file 3: Figure S2). Most ARF proteins contain a highly conserved N terminal DBD composed of an unknown subdomain, a plant specific B3-type subdomain, an ARF subdomain and nuclear localization signal (NLS) [55]. In the present work, motif 1–4, 6–9, 12, and 13 correspond to the N-terminal DBD in all of 14 PsARFs. Among these motifs, the biological significance of motif 3 and 4 remains uncharacterized (Additional file 3: Figure S2). However, motif 2–1-9 and 6–13–8-12-7 orderly constituted the B3-type and ARF subdomain, respectively (Fig. 1a and b). Interestingly, 3 out of the 14 PsARFs (PsARF10, 16 and 17) exhibited an additional short segment of amino acids between the B3-type and ARF subdomain (Fig. 1a). At the end of the DBD domain, all of the PsARFs contain a motif 11, which correspond to a conserved putative NLS (Fig. 1a). In addition, the motif 14, 10 and 5 orderly formed the CTD region consisting of subdomain III and IV (Fig. 1b).The predicted protein structures of PsARF3, 8 and 17 are lacking dimerization subdomain III and IV (Fig. 1a), while PsARF10 and 16 have a truncated CTD (only segmental subdomain III) (Fig. 1a and Additional file 3: Figure S2). It was reported that these non-conserved MD in at least some *Arabidopsis* ARFs function as transcriptional activation (Q-rich) or repression domains (P, S and T-rich) [45]. By component analysis in the amino acid of 14 PsARF proteins, the middle region of PsARF1–6, 10–11, and 16–17 is rich in P or S, whereas the middle region of PsARF7 and 19 is rich in Q (Fig. 1a and Additional file 4: Table S2). Additionally, the middle region of PsARF 8 and 9 is enriched in QS and Asparagine (N), respectively (Fig. 1a). Overall, the conserved motifs were found in PsARF proteins, indicating a strongly functional constraint during the course of evolution.

### Phylogenetic analysis of PsARF proteins

**Fig. 2:**
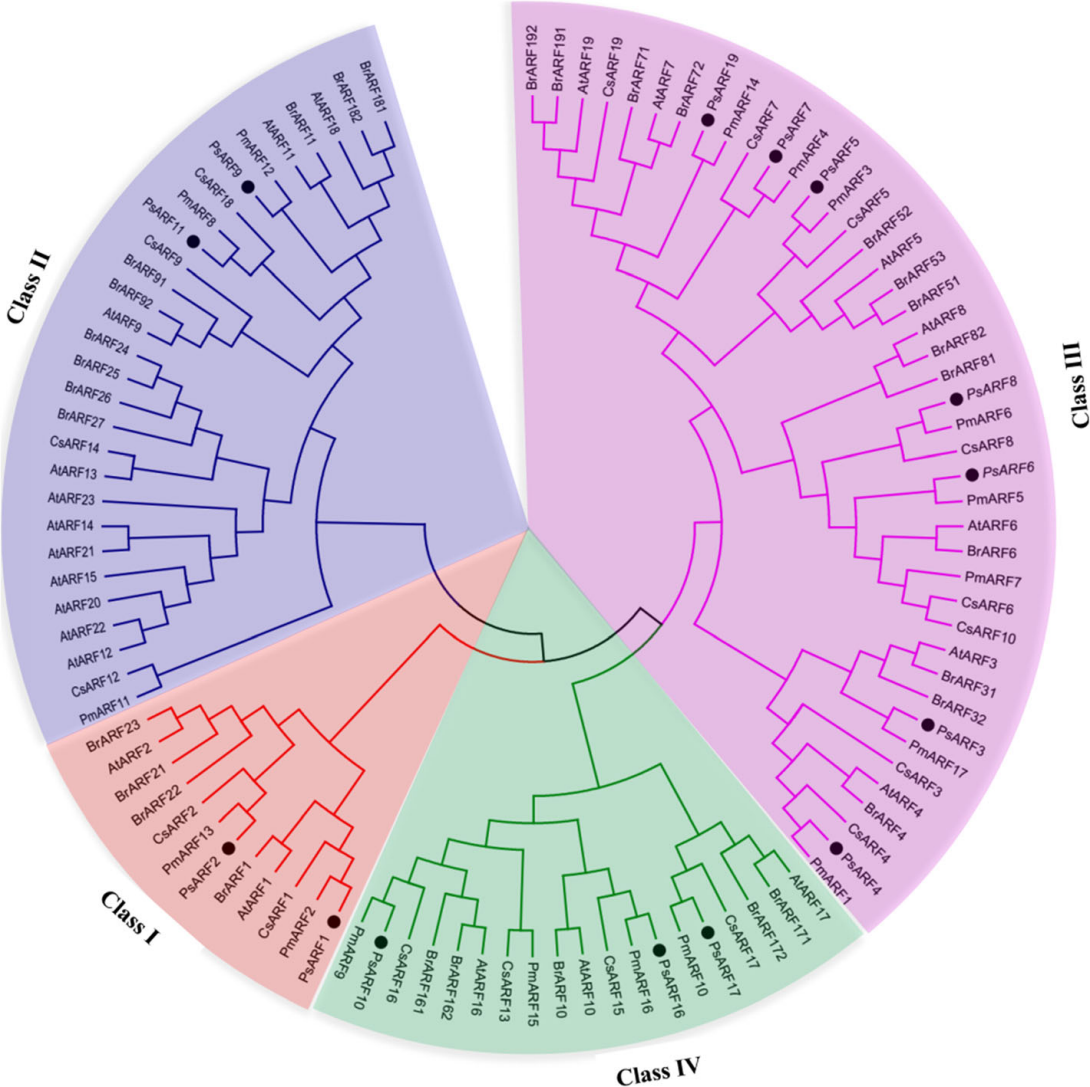
Phylogenetic analysis of ARFs from *Arabidopsis*, *B. rapa*, *C. sinensis*, *P. mume*, and *P. sibirica* by using Neighbor-joining method. Groups of genes are represented by color arcs. *At*: *Arabidopsis thaliana*, *Br*: *Brassica rapa*, *Cs*:*Citrus sinensis*, *Pm*: *Prunus mume, Ps*: *Prunus sibirica*

To investigate the evolutionary relationship between PsARFs and those in other species, a neighbor-joining tree was generated based on alignments of the complete protein sequences of 14 PsARFs, 23 AtARFs, 31 BrARFs, 19 CsARFs, and 17 PmARFs. The results showed that all the ARFs could be grouped into four major clusters (I, II, III, and IV) based on their phylogenetic relationship (Fig. 2). 14 PsARFs were distributed among the four clusters, for example, Cluster I included PsARF1 and 2; Cluster II included PsARF9 and 11; Cluster III included PsARF3, 4, 5, 6, 7, 8, and 19; and Cluster IV included PsARF10, 16, and 17 (Fig. 2). Interestingly, our phylogenetic analysis unambiguously established the orthologous relationship between PsARF and PmARF proteins, indicating that the organization of the PsARF proteins is very similar to that of the PmARF proteins. These results imply that PsARF and PmARF proteins are close relative, and indeed *P. mume* belongs to the same family Rosaceae with our experimental material (*P. sibirica*).

### Expression of *PsARF* genes in developing mesocarp and kernel

**Fig. 3:**
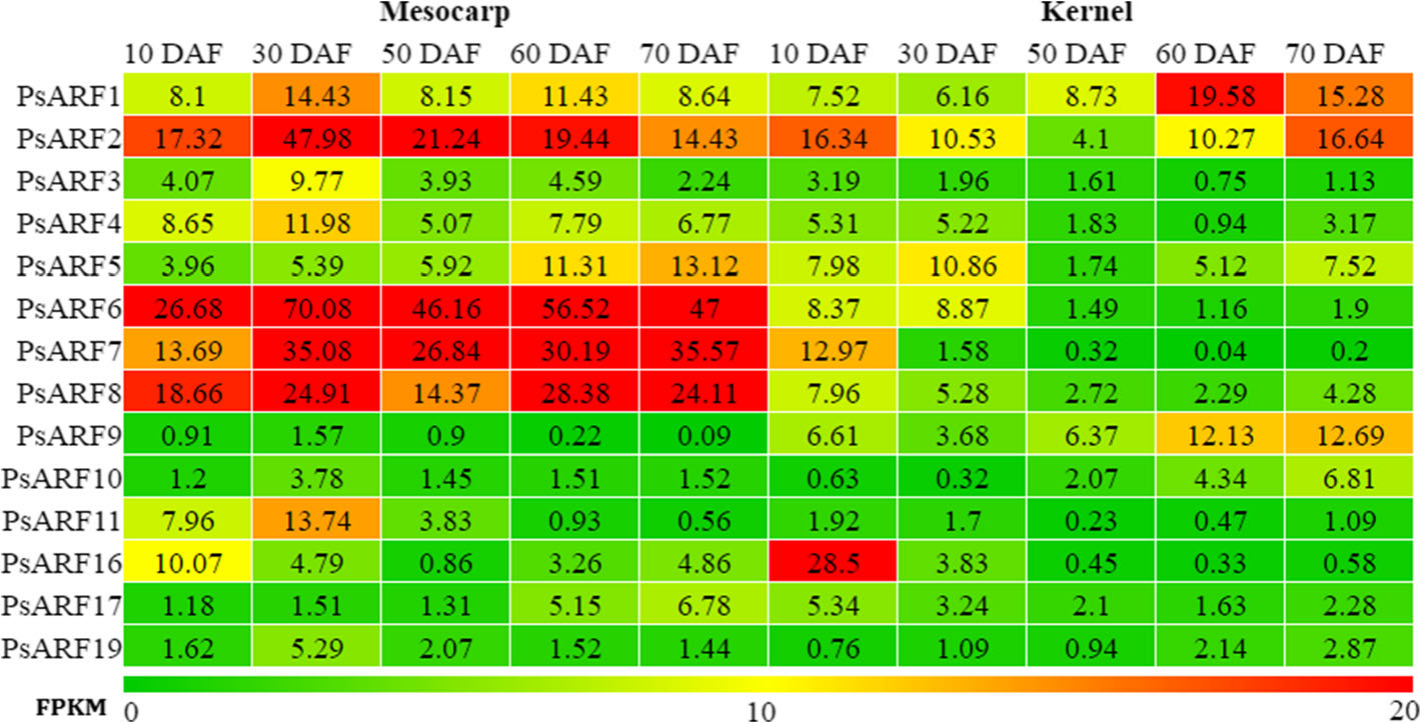
The expression levels (FPKM) of *PsARF* genes in developing SAM and SAK. The data in heatmap showed FPKM values

**Fig. 4:**
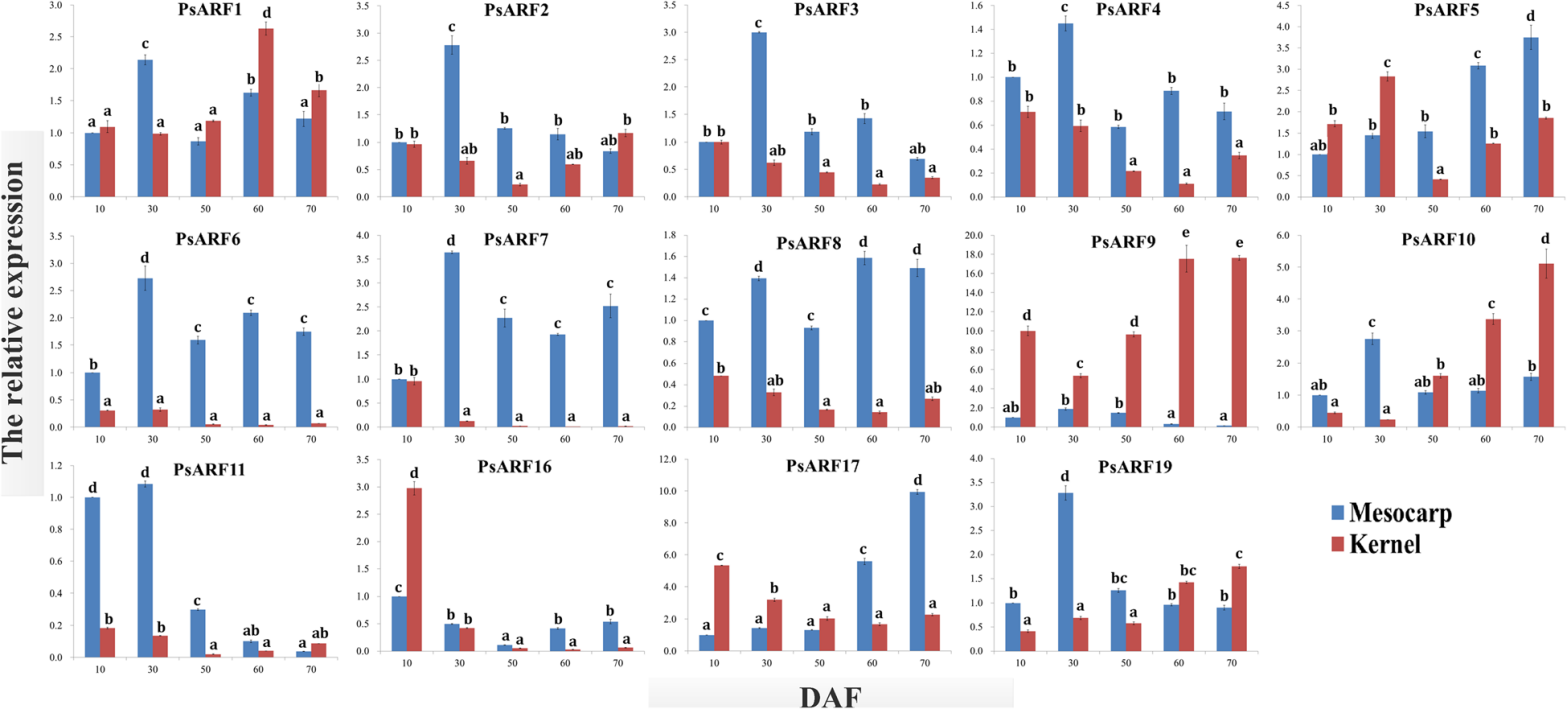
The qRT-PCR analysis of *PsARF* genes in developing SAM and SAK. The relative expression levels were calculated as 2^-△△Ct^, and the expression data of SAM at 10 DAF served as control. Data are means ± SE of three separate measurements

To better understand the expressional characteristics of each *PsARF* gene in developmental process of SAM and SAK, the expression profiles of *PsARF* genes were investigated by qRT-PCR and FPKM. In this study, the expressions of all 14 *PsARF* genes could be detected in both the SAM and SAK (Figs. 3 and 4).

During the development of SAM, 14 *PsARF* genes presented complicated expression patterns (Figs. 3 and 4). 10 *PsARF* genes (*PsARF1–8*, *11* and *16*) were highly induced (value >10) at specific stages of SAM development, while *PsARF9*, *10*, *17* and *19* showed low expression (value <10) at all stages of SAM development (Fig. 3). Among the highly expressed genes, most of *PsARF* genes, such as *PsARF1*–*4*, *11* and *16*, showed transcript accumulation before 30 DAF, whereas only *PsARF*5 were significantly up-regulated at late development (60–70 DAF) of SAM (Fig. 3), as was in accordance with our qRT-PCR results (Fig. 4). Notably, *PsARF2* and *6–8* exhibited high expression levels (value >10) at all stages of mesocarp development (Fig. 3).

As for the development of kernel, *PsARF1*, *2*, *5*, *7*, *9* and *16* demonstrated significant induction (value >10) at specific stages of SAK development (Fig. 3). Our qRT-PCR analysis revealed that *PsARF7* and *16* were significantly up-expressed at 10 DAF, whereas *PsARF1* and *9* were abundantly transcribed at 60–70 DAF (Fig. 4). Additionally, *PsARF2* and *5* showed down-regulated transcript at 50 DAF (Figs. 3 and 4). It is worthy to note that more *PsARF* genes, including *PsARF3*, *4*, *6*, *8*, *10*, *11*, *17* and *19*, showed low expression (value <10) at all developmental stages of SAK in comparison to SAM (Fig. 3).

In a comparison of the expression profiles of *PsARF* genes at different developmental stages of SAM and SAK, *PsARF4*, *6*, *7, 8*, and *11* exhibited specifically higher expression in SAM, while *PsARF9* were transcribed more strongly in SAK (Figs. 3 and 4). Also, *PsARF 10*, *17* and *19* showed low expressions (value <10) at all stages of SAM and SAK development (Fig. 3). Interestingly, *PsARF16* shared a similar pattern of mRNA accumulation in developing SAM and SAK (Figs. 3 and 4), implying that *PsARF16* may play similar roles in early development of SAM and SAK. Together, our expression data showed a high variability in transcript abundance of the *PsARF* genes between SAM and SAK (Figs. 3 and 4), probably indicating the diversified functions of the *PsARF* genes in controlling SAM and SAK development.

### Co-expression network analysis of *PsARF* genes

**Fig. 5:**
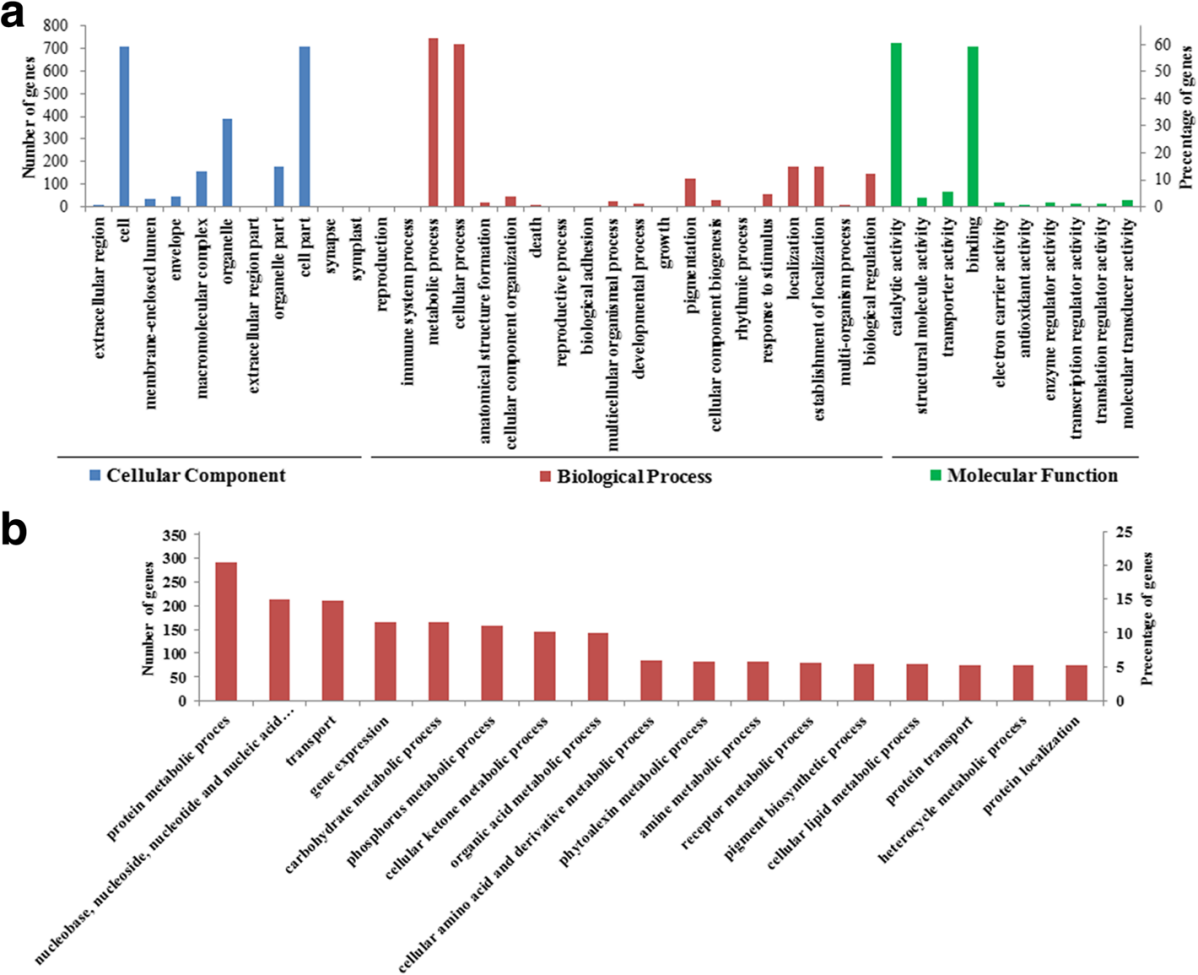
Histogram presentation of Gene Ontology classification. **a** The results are summarized in three main categories: biological process, cellular component, and molecular function. **b** The major subcategories in metabolic process. The y-axis on the left indicates the number of genes, and the y-axis on the right means the percent of genes in a category

By using previous expression data from *E. guineensis* [4], *P. sibirica* [31], *B. napus*, *R. communis*, and *E. alatus* [44], we attempted to establish the co-expression network of *ARF* genes. A total of 8 *ARF* genes (homologous *PsARF1*, *2*, *6*, *8*, *9*, *11*, *16* and *17*) were shared by the above 5 plant species, suggesting that these *ARF* genes may be constitutively expressed. Using WGCNA software, the resulting 79, 1265, 9, 35, 2 and 148 genes were identified to be involved in the co-expression network of the homologous *PsARF1*, *8*, *9*, *11*, *16* and *19*, respectively (Additional file 5: Table S3). To fully explore the biological functions of these *ARF*-correlated genes, GO annotation was performed. The functional terms of 1514 *ARF*-correlated genes, covering 41 subcategories, were assigned to 3 main GO categories, for example, “GO:0044464 cell part”, “GO:0008152 metabolic process” and “GO:0003824 catalytic activity” had the highest frequencies in cellular components, biological processes and molecular function, respectively (Fig. 5a and Additional file 6: Table S4). Notably, most of *ARF*-correlated genes were identified to be related to cell metabolism, and thus we further explored the detailed category. “GO:0044260 protein metabolic process”, “GO:0006139 nucleobase, nucleoside, nucleotide and nucleic acid metabolic process”, “GO:0006810 transport”, “GO:0010467 gene expression” and “GO:0005975 carbohydrate metabolic process” were the top 5 subcategories (Fig. 5b). Moreover, these *ARF*-correlated genes participated in phosphorus, ketone, lipid, amino acid derivative and other metabolic processes (Fig. 5b), suggesting that ARFs may play a crucial role in several metabolic processes.

## Discussion

It is known that auxin is a key signaling molecule for most organogenesis and patterning processes during plant development. In plants, the ARFs could directly bind to the AuxRE of down-stream target genes and mediate their transcription involved in various biological processes [6, 25]. Recently, the characterization of *ARF* genes has been reported in *Arabidopsis* [18], *O. sativa* [47], *Z. mays* [28], *P. trichocarpa* [20] and *Gossypium raimondii* [42], but not in Siberian apricot, a novel and important woody oilseed species. In this study, 14 *ARF* transcription factor genes with a complete ORF were identified in *P. sibirica* according to transcriptomic data form different developing fruits [31] and different tissues [9].

Generally, the detailed information about the protein domains is helpful for understanding the function of the corresponding gene. It has been reported that most ARF proteins consist of an N-terminal B3-type DBD, a C-terminal CTD, and a variable MD that functions as an AD (rich in Q) or RD (rich in PST) [12]. As a typical ARF-type structure, the DBD domain is important for the recognition of AuxRE (TGTCTC) in the promoters of auxin-responsive genes [55]. Indeed, the DBD was identified in all 14 PsARF proteins (Fig. 1a), suggesting that DBD may be essential region for the function of PsARF proteins. It was reported that some ARFs exhibited an additional short segment of amino acids between the B3 and ARF subdomain in *Arabidopsis* [18] and *P. trichocarpa* [20]. Such a feature has also been observed in PsARF10, 16 and 17 proteins (Fig. 1a). At the end of the DBD domain, all the PsARF proteins contain a monopartite NLS, which has been experimentally confirmed to be able to direct the ARF proteins into the nucleus by a synthetic green fluorescent protein fusion assay in rice [40]. For the conserved structure of CTD, the region is directly involved in the hetero-dimerization of ARF and Aux/IAA proteins [23]. In this study, 5 PsARFs (PsARF3, 8, 10, 16 and 17), which lack a partial or complete CTD (Fig. 1a), should consequently be insensitive to auxin [13]. However, loss of CTD could also have consequences on the interaction of ARFs with other transcription factors [12]. The amino acid composition analysis of MD sequence in all 14 PsARF proteins revealed that PST rich regions were found in the middle regions of PsARF1–6, 10–11 and 16–17 (Fig. 1a), suggesting that these PsARFs might function as transcriptional repressors. In addition, PsARF7 and 19 harbour a Q-rich middle region (Fig. 1a), implying that PsARF7 and 19 are possibly transcriptional activators. Certainly, the specific regulatory role of PsARFs is misleading if without further experiments of functional verification.

We also built a phylogenetic tree to analyze the relationship of ARF families between *Arabidopsis*, *B. rapa*, *C. sinensis*, *P. mume*, and *P. sibirica* (Fig. 2). The result revealed that ARFs are distributed into four major clusters (I, II, III, and IV), which was similar to the previously phylogenetic classifications of ARFs in *E. grandis* [55], banana [52] and *Arabidopsis* [34]. Generally, the close relative species should expect to have similar structures in the same protein family. Indeed, ARFs from the *P. sibirica* were more closely related to those from *P. mume* than those from *Arabidopsis*, *B. rapa* and *C. sinensis* (Fig. 2)*.*

Fruit (including mesocarp or seed) development is a complex interplay of cell division, differentiation and expansion that occurs in a temporally and spatially coordinated manner in the reproductive organs [15]. To assess the function of the *PsARF* genes in developing SAM and SAK, we investigated the expression profiles of *PsARF* genes by FPKM and qRT-PCR. Although the expressions of all 14 *PsARF* genes could be detected in both the SAM and SAK, different *PsARF* showed distinct temporal and spatial expression pattern in developing SAM and SAK (Figs. 3 and 4). In *Arabidopsis*, ARF1 may act with ARF2 to control aspects of maturation and senescence [10]. The homologous *PsARF1* was significantly expressed at 60–70 DAF of SAK (Fig. 4). This finding, together with the involvement of highly *PsARF1*-correlated *ETHYLENE INSENSITIVE 4* gene in growth and development of *Arabidopsis* [26], suggested that *PsARF1* might play crucial roles in late SAK development, such as the acquisition of dormancy and desiccation tolerance. In addition, the high expression of *PsARF 9* was characteristically transient at 60–70 DAF of SAK (Figs. 3 and 4). It has been reported that *AtARF9* act in suspensor cells to mediate hypophysis specification in *Arabidopsis* [35]. One of *PsARF9*-correlated genes was also identified to code a RCD1-like protein involved in cell differentiation [50].

The mutation analyses revealed that *AtARF2* is a general repressor of cell division to regulate seed size and weight [38]. Indeed, *PsARF2* exhibited high expression levels (value >10) at all developmental stages of SAM (Fig. 3), implying potential influence of *PsARF2* in fruit size. Additionally, high expression levels (value >10) of *PsARF6*, *7* and *8* at all developmental stages of SAM (Fig. 3) suggested that these genes might be involved in mesocarp development, as was reported that *ARF8* regulate fertilization and fruit development in *Arabidopsis* [14]. By GO annotation in biological process, we identified most genes correlated with *PsARF8* genes were widely involved in cellular metabolic and regulatory process, including carbohydrate, amino acid, organic acid and lipid metabolism. Interestingly, the *SlARF4* that is highly homologous to *PsARF8* was identified to be involved in the control of sugar metabolism during tomato fruit development [36]. Thus, it is tempting to speculate that *PsARF8* may be a master transcriptional factor participated in the regulation of carbohydrate metabolism during SAM development.

Previous investigation in stem cell of *Arabidopsis* showed the responsible of *AtARF16* for pattern specification process that results in the creation of defined areas or spaces within an organism to which cells respond and eventually are instructed to differentiate [8]. In this study, specifically transcriptional accumulation of *PsARF16* was found at early development (10 DAF) of SAM and SAK (Figs. 3 and 4). Remarkably, *PsARF16*-correlated *REVEILLE 6* gene, as one of circadian rhythm regulator, could inhibit plant growth in *Arabidopsis* [16]. Thus, the abundantly similar transcripts of *PsARF16* at early development of SAM and SAK may help to a series of programmed cell divisions and the basic architecture of the plant. In addition, the high expression of *PsARF11* was characteristically transient at early SAM development (Figs. 3 and 4). By function annotation, two *PsARF11*-correlated genes (*LEUNIG_HOMOLOG* and *LEUNIG*) regulate mucilage extrusion, mainly composed of pectin, required for mucilage maturation during *Arabidopsis* fruits development [5]. Thus, *PsARF11* may be involved in cell wall formation.

Here, the observed low transcript of *PsARF10*, *17* and *19* both in developing SAM and SAK (Fig. 3) indicated that these genes are not likely to play important roles in SAM and SAK development. As in the case of *Arabidopsis*, no phenotypic defects were reported for *ARF10* and *19* single mutants [34]. Summary, lineage-specific expression of *PsARF*s may create a pattern enabling different developmental auxin responses required for normal SAM and SAK development.

## Conclusions

As ARF transcriptional factor is known to be implicated in regulation of fruit and seed development, it is important to understand their structure, transcription and regulation. Based on our previous transcriptomic data, we identified 14 *PsARF* genes with a perfect ORF, and analyzed their phylogenetic relationship, gene structure and protein motifs. The current work has contributed to an increased knowledge of the *ARF* gene family in *P. sibirica*. The expression profiles and co-expression networks of *PsARF* genes will provide a fundamental basis for in-depth experimental studies of *ARF* function in Siberian apricot.

## Additional files

Additional file 1: Table S1. Primer sequences used for real-time PCR. (DOCX 19 kb)

Additional file 2: Figure S1. The electropherogram of 14 *PsARF* genes. (TIF 77 kb)

Additional file 3: Figure S2. Amino acid sequence alignments of 14 conserved motifs. (TIF 219 kb)

Additional file 4: Table S2. Components analysis in the amino acid of middle regions of 14 PsARF proteins. (XLSX 14 kb)

Additional file 5: Table S3. Co-expression analysis of *ARF* genes. (XLSX 74 kb)

Additional file 6: Table S4. Function and GO annotation of co-expression genes correlated with *ARF*s. + means the correlation of co-expressed genes with corresponding *ARF*, and – means no correlation of co-expressed genes with corresponding *ARF*. (XLSX 198 kb)

## Abbreviations

AD: Activation domain
ARF: Auxin response factor
Aux/IAA: Auxin/indole-3-acetic acid
AuxRE: Auxin response element
CTD: Carboxy-terminal dimerization domain
DBD: DNA-binding domain
FPKM: Fragment Per Kilobase of exon model per Million mapped reads
GO: Gene Ontology
MD: Middle domain
MEME: Multiple Expectation Maximization for Motif Elicitation
N: Asparagine
NLS: Nuclear localization signal
ORF: Open reading frame
P: Proline
Q: Glutamine
RD: Repression domain
S: Serine
SAK: Siberian apricot kernel
SAM: Siberian apricot mesocarp
T: threonine
