# Supplementary material for: Identification of AUXIN RESPONSE FACTOR gene family from Prunus sibirica and its expression analysis during mesocarp and kernel development
Source: BMC Plant Biol. 2018 Jan 24;18:21. doi: 10.1186/s12870-017-1220-2 (PMC5784662; doi:10.1186/s12870-017-1220-2)
Supplement: Supplementary file 1 — Primer sequences used for real-time PCR. (DOCX 19 kb) [file 12870_2017_1220_MOESM1_ESM.docx]

**Supplementary Table S1 Primer sequences used for full-length clone and real-time PCR**

|  | **Genes** | **Forward primer** | **Reverse primer** |
| --- | --- | --- | --- |
| **full-length clone** | PsARF1 | TTCAGGGGTGTGAGAACATTTGT | TCAGTATGCAGGTCCCACAATG |
|  | PsARF2 | CTCTGAGCTTTTGTAGAAATGACGT | TACCTAAAAATCTTCAGAATCAGGC |
|  | PsARF3 | TTCCACATGGGGGGTCTAATC | ATTGTATCATTGCTATCCTTGTA |
|  | PsARF4 | TGTTTACTGGGTTGTGCTTTCTG | TTAGACCCTGATTACTGTTGGGG |
|  | PsARF5 | CATGATAATGGGCTCAGTTGAGG | TGGCACGGTCAGAGGCAGC |
|  | PsARF6 | GGCTTTCGACGTTTTTGAATC | ACCGGTTAAGTCGTTCAATACTCGA |
|  | PsARF7 | ATGAAGCCGCCCGCGAACGGAGCCG | GTCCACTACATTAGGCATTCCCACC |
|  | PsARF8 | ATATGAAGCTTTCAACATCAGGGTT | CTTTCATCTCACACCTTGACAAATG |
|  | PsARF9 | CGAGTTTCAGCGATGGCGAATC | GATGTTAGTCCGAGCTTGTTACGG |
|  | PsARF10 | GCCATACAAGGAATTTTTATGCTGT | TGTGGAACTCAGCCCATATAAAGTC |
|  | PsARF11 | GAGCGTTTGAATGGCACACCTT | ATCATGTTTCACATCTATGCTCGGA |
|  | PsARF16 | CATCCGGGTACCTTTTGAAATTG | GACTCCATGCAGAGTTCAAAATTCA |
|  | PsARF17 | CAAAATGCCTCCTACGCCTCATC | TGTTGTAGCAGCCACCAGTAGTTCA |
|  | PsARF19 | TAGACATATCATTCAAATCTCAAGC | CTTTACCGATTAAATGAGGCTG |
| **qRT-PCR** | PsARF1 | GTTGGACGAGCCGTAGATTT | CACAGAGCTCACCCTCAATATC |
|  | PsARF2 | AATGGGTCACCTCACCATAAC | CAGTTTATCCGGCTCACTAACA |
|  | PsARF3 | TGAGGTGCCTTGATCATTCC | CCAGTTATCAGCCCAGTGTATC |
|  | PsARF4 | CTTGATGGTCAGATGGGATGAA | GCTCAAAGGTGGGAGAGAAA |
|  | PsARF5 | GAGCCAGGGTGTTGTGATAA | CCCAAGAATCCAGAGTGTAGTG |
|  | PsARF6 | CCTGGCCGGAGTTTGTAAATA | CTGAGTTGGGACTGACTTTAGG |
|  | PsARF7 | GTGGAGCTGTAGGGAGATCTAT | GGTCCTCTAACTGTCCCTCTAT |
|  | PsARF8 | GTCCTGAAGGGAGTGGAAATC | CTGTGCATGTGCATACTTTGG |
|  | PsARF9 | GAACCACTGAAGGACGTGTTA | CAACTGTCCAGGTTTGCTTTC |
|  | PsARF10 | AACCCAGACTATTCCCTTGTTAG | CCTGTATGCCCGCTGATATT |
|  | PsARF11 | ATGTCGTGTGGTGGTAAAGG | CTGGTGATGCCTCTGAGATTAC |
|  | PsARF16 | CTCTCGGTTCTTGGCTCTTATG | CCTGTCACATCCCGGTAAAG |
|  | PsARF17 | GTACAATGCAACTGAAGGTGTG | ACAGCTGAGGCTCTTTGATATT |
|  | PsARF19 | CAAAGGGACCAGACCAGATAAA | TGTGCTAGCATCCAGACAATAC |
|  | CYP | CAACGGATCTCAGTTCTTCGTCTGC | GACCCAACCTTCTCGATGTTCTTCA |
|  | UBC | GAGACCAGCAATAACCGTGAA | TCTTGTACTCCGTGGCATCCT |
