# Supplementary figures and images for: Identification of AUXIN RESPONSE FACTOR gene family from Prunus sibirica and its expression analysis during mesocarp and kernel development
Source: BMC Plant Biol. 2018 Jan 24;18:21. doi: 10.1186/s12870-017-1220-2 (PMC5784662; doi:10.1186/s12870-017-1220-2)

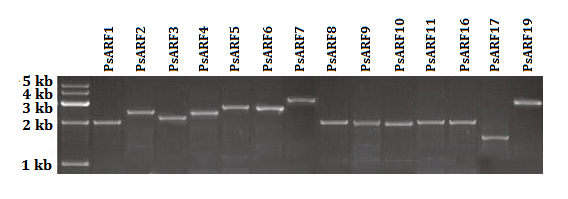

Supplement: Supplementary file 2 — The electropherogram of 14 PsARF genes. (TIF 77 kb) [file 12870_2017_1220_MOESM2_ESM.tif]

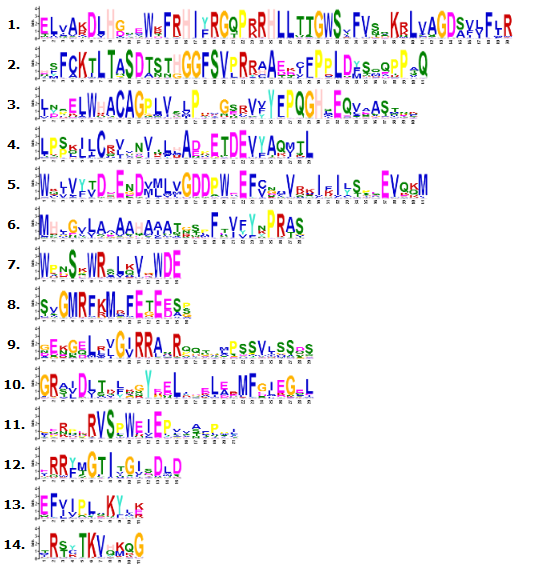

Supplement: Supplementary file 3 — Amino acid sequence alignments of 14 conserved motifs. (TIF 219 kb) [file 12870_2017_1220_MOESM3_ESM.tif]
